# Regeneration of immature incisor using platelet rich fibrin: report of a novel clinical application

**DOI:** 10.1186/s12903-023-02759-9

**Published:** 2023-02-02

**Authors:** Jishnu Krishna Kumar, Padmapriya Surendranath, Rajalakshmanan Eswaramoorthy

**Affiliations:** 1 Department of Public Health Dentistry, Saveetha Dental College and Hospitals, Saveetha Institute of Medical and Technical Sciences (SIMATS), Chennai, India; 2 Tulsi Dental Care Advanced Paediatric and Family Dental Clinic, Palakkad, India; 3 Department of Applied Chemistry, School of Applied Natural Science, Adama Science and Technology University (ASTU), PO. 18888, Adama, Ethiopia; 4 Department of Biomaterials, Saveetha Dental College and Hospitals, Saveetha Institute of Medical and Technical Sciences (SIMATS), Chennai, India

**Keywords:** Trauma, Regenerative endodontics, Nonvital tooth, Tooth apex

## Abstract

**Background:**

Endodontic treatment of young permanent teeth with necrotic pulp presents a clinical challenge for the dentist, and conventional endodontic treatment will result in tooth fracture along with a poor prognosis. Regenerative endodontics is a new protocol that has been advanced in the last decades for managing immature permanent teeth. Rare successful management of immature permanent incisors using platelet-rich fibrin is a technique-sensitive procedure.

**Case presentation:**

A 08 years 04 months old female reported the chief complaint of pain in the upper front tooth region for one week. A blunderbuss canal was identified on radiographic examination, and revascularization using platelet-rich fibrin was planned and adopted. After the treatment, apical closure and root lengthening were noted without complications during subsequent follow-ups. Complete periapical healing with greater than 1.5 cm of dentinal thickness was noted.

**Conclusion:**

Revascularization can be considered a viable treatment option for immature nonvital permanent teeth; with advancements in regenerative medicine and clinical practices, revascularization therapies could be developed as a novel mode of treatment in non-vital and dental traumatic cases.

## Introduction

Endodontic treatment of young permanent teeth with long stranding necrotic pulp is often managed with questionable prognosis—incompletely developed teeth managed by conventional root canal therapy [1]. Immature necrotic teeth treated by apexification or an apical artificial barrier of MTA (Mineral Trioxide Aggregate) or Biodentine carry a greater fracture risk because of arrested root development. Also, traditional apexification using calcium hydroxide carries the risk of proteolysis and dehydration, especially in immature non-vital teeth [2, 3].

Over the decades, there has been a paradigm shift in regenerative endodontic treatment (RET) philosophy. The RET procedures carry the advantage of continued physiological root development and utilize the concept of tissue engineering to restore the root canals and the peri-radicular area to a healthy state. The procedures use scaffolds, growth factors, and stem cells to promote continuous optimum root structure development and were first reported by Iwaya et al. (2001) [4]. The factors leading to new epithelial in-growth into the non-vital immature permanent tooth are conclusively unexplored; the in-growth can be prevented only through technique-sensitive procedures in non-vital teeth utilizing regenerative techniques. Careful manipulation of such cases with RET would effectively improve the child’s quality of life and could provide a paradigm shift to pedodontic regenerative therapies [5]. Moreover, failures in regenerative endodontic treatment are high and retreatment of such failed RET could be challenging. Considering such difficulties, a successfully established RET is essential at the first attempt. The commonly noted reasons for the failure of regenerative therapies are root resorption and persistent infection, within six months of treatment as deciphered by Lee et al. [3, 6].

One of the latest innovations in dentistry is platelet concentrates for tissue engineering; being autologous and easy to prepare, platelet concentrates comprise a high concentration of growth factors. Platelet-rich fibrin (PRF) has been shown to promote better healing properties, over platelet-rich plasma [7]. PRF contains an autologous leukocyte-platelet-rich fibrin matrix and a tetra molecular structure with cytokines, platelets, and stem cells. It acts as a biodegradable scaffold that favors microvasculature development and epithelial cell migration; also helps in the sustained release of growth factors (1–4 weeks) [8]. Further, PRF needs less preparation time and no additives; though the gel consistency of PRF makes it difficult during placement, following standard placement protocols and clinical prowess may improve the prognosis of the regenerative technique [7, 9].

Here we are reporting a rare successful case of revascularization in a traumatized nonvital young permanent tooth using PRF.

## Case presentation

**Fig. 1:**
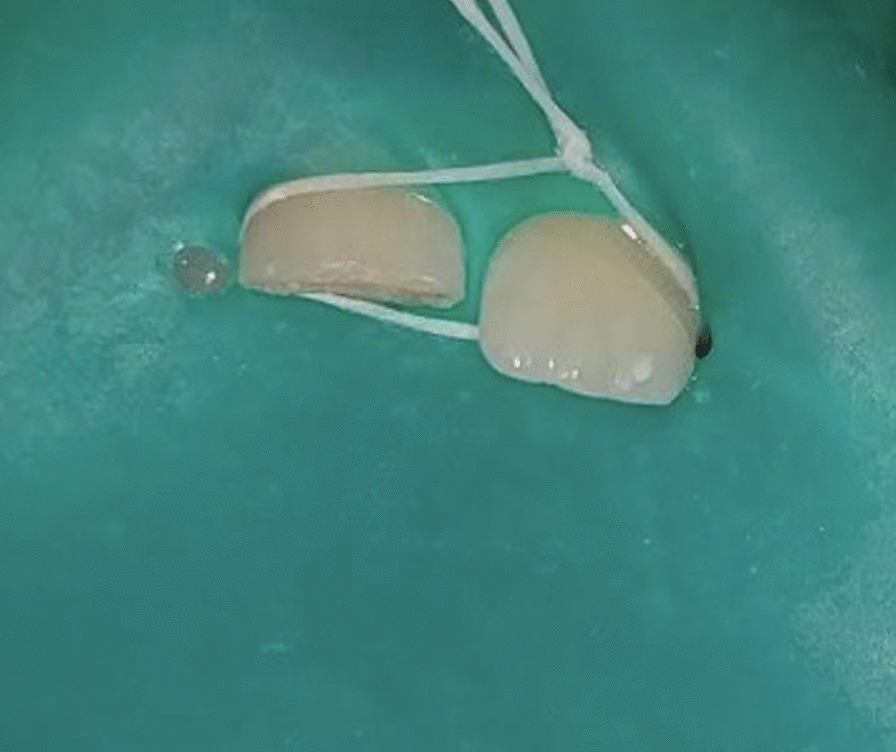
Preoperative clinical photograph of non-vital permanent anterior tooth

**Fig. 2:**
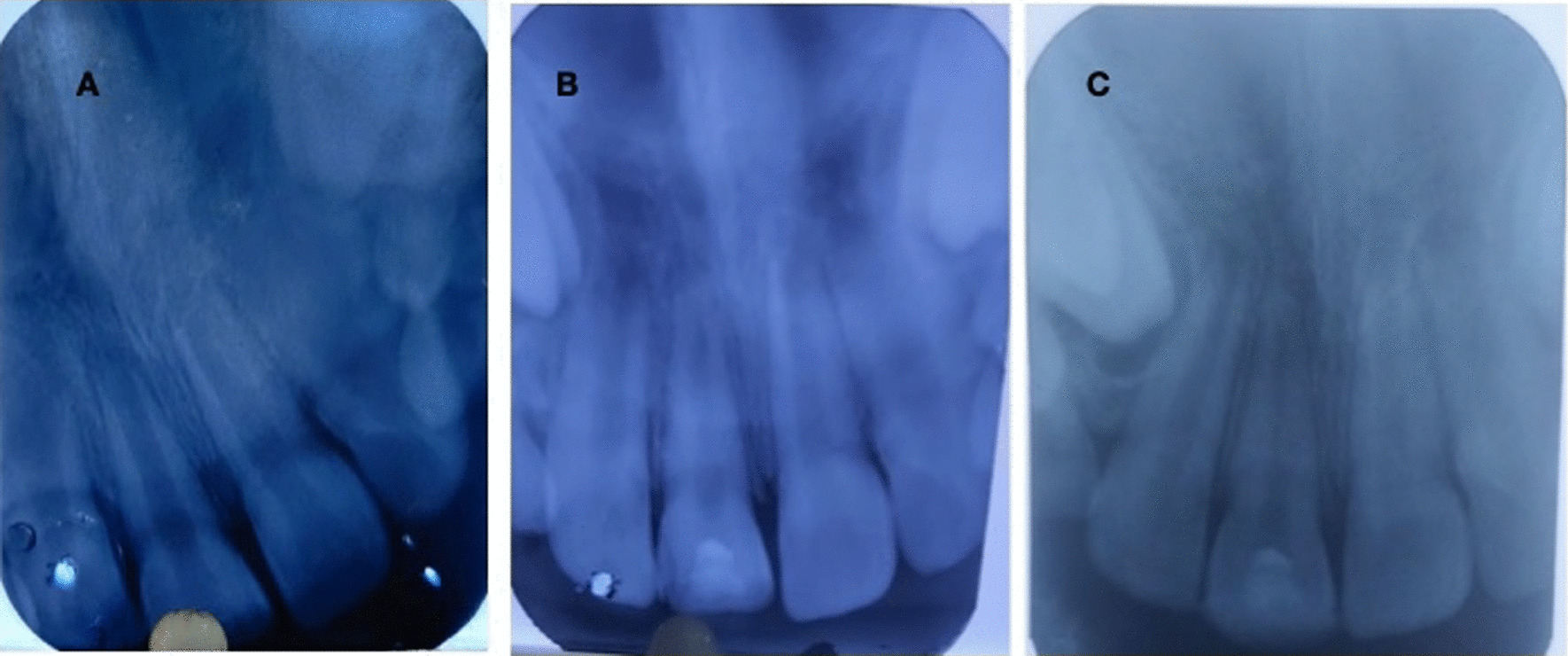
(**A**) Preoperative radiograph of non-vital 11 (Blunderbuss canal/open apex) (**B**) Radiograph at six months (increase in Dentine thickness and root end closure) (**C**) Radiograph at nine months (apical close closure and root canal obliteration)

An eight-year four-months-old female child reported to the outpatient unit of the Pedodontics department of a tertiary dental care teaching and research hospital, with the chief complaint of pain in the upper front tooth region. The patient gave a history of trauma (self-fall) six months back. The child’s medical history was non-contributing; a class II fracture was noted at about 11 (Fig. 1). Radiographs were taken using bisecting angle technique and size 0 intraoral periapical radiograph (22 mm × 35 mm) for radiographic diagnosis. The periapical radiograph revealed a fracture involving the pulp with blunderbuss canals (Fig. 2). On further examination, no response was elicited after electric pulp testing, conclusive of the tooth being nonvital; also, dental caries was noted concerning 84 and 75. The decision to do revascularization using PRF was taken after correlating clinical and radiological findings with careful examination and as the patient was young. The inclusion criteria for the case were considered to be a young permanent tooth with an open apex and thin dentinal walls. Vital young permanent teeth and those teeth that cannot be restored were excluded from the study. Consensus-based Clinical Case Reporting Guidelines (CARE) were used as standard guidance to report the case flow and process of treatment (Fig. 6) [10].

### Ethical clearance and consent

The patient’s and parent’s informed consent was obtained as it involved drawing the patient’s blood. Further, all the details, such as investigations, clinical procedures, complications, and alternative options were explained to the parents, as per the patient information sheet. Ethical approval was obtained from Institutional Review Board in Chennai, India, without any delay as the procedure did not involve the experimental of any armamentarium and the safety of the technique had been established.

### First treatment visit

In the first appointment, the tooth was identified and anesthetized by buccal and palatal infiltrations by administering 0.5 ml 2% Lidocaine with 1:80,000 adrenaline. Further, isolation was carried out with a single-use Rubber dam (green) with a #26A clamp. Access opening was done to gain straight-line access to the pulp chamber using #4 round diamond bur, and no bleeding was noted; minimal instrumentation was done to remove dead tissues and dentine ledges. The pulp chamber was irrigated with 0.09% normal saline (20 ml). Working length was taken using an 80 K file. The canals were dried using sterile paper points and Triple antibiotic paste was placed. A sterile cotton pellet was placed and sealed temporarily using Zinc Oxide Eugenol cement. The triple antibiotic paste is considered the reference standard for regenerative endodontics and was placed to disinfect the canal as per standard regenerative protocol [11]. and the child was recalled after two weeks.

### Second treatment visit

**Fig. 3:**
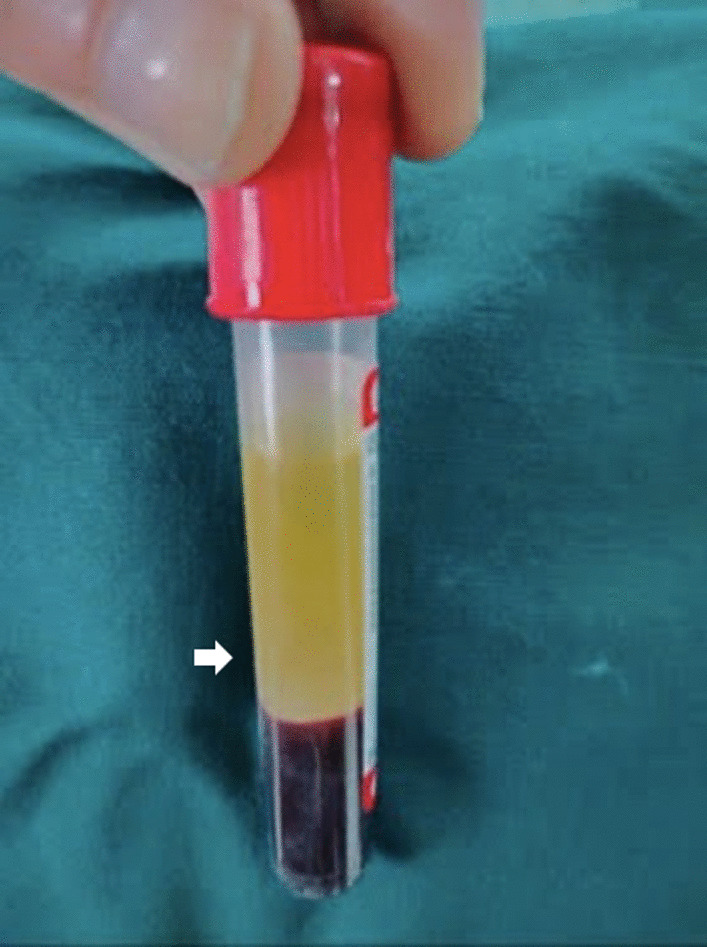
Platelet-rich Fibrin seen after centrifuge

In the second appointment, blood was drawn before the procedure, and PRF was prepared as per standard preparation protocol (Fig. 3); squeezed in gauze to remove excess fluid, and cut into small linear strips for easy placement [12]. The triple antibiotic paste was removed from the canal with thorough irrigation with normal saline. Bleeding was induced, and PRF was placed in the canal. The canal orifice was sealed with mineral trioxide aggregate (MTA); a wet cotton pellet was placed over it and sealed with zinc oxide eugenol temporary cement. Further, a postoperative radiograph was done; after 24 h, the patient was recalled, the pellet was removed, and type II glass ionomer cement was placed. The composite filling was done to reform aesthetics, and post-operative instructions were given. The child was recalled in the sixth, ninth, and twelfth months and was evaluated clinically and radiographically.

### Follow-up examination

**Fig. 4:**
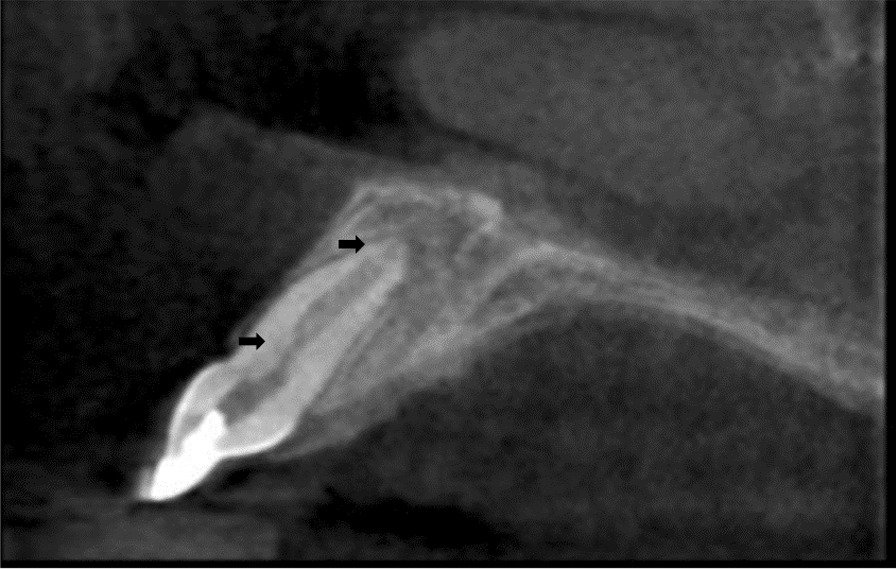
CBCT image at 12 months of 11 (three-dimensional apical closure)

**Fig. 5:**
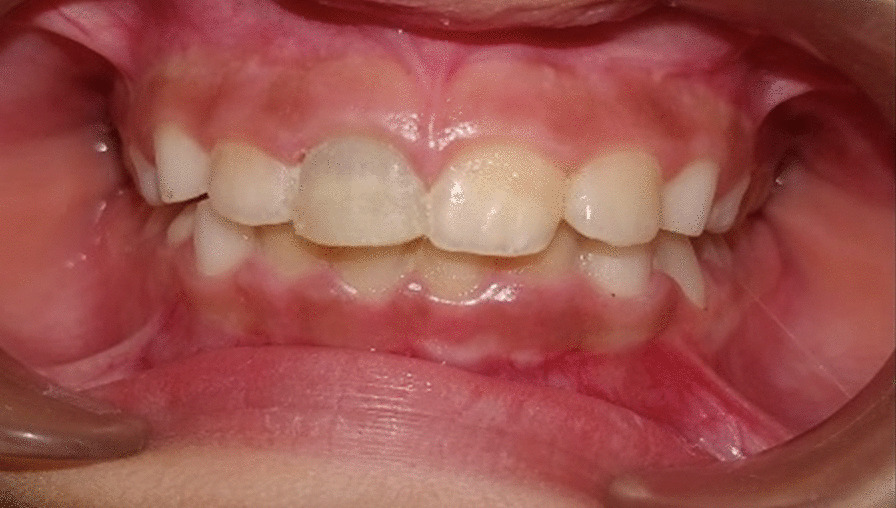
Postoperative clinical photograph at 12 months (clinically and morphologically asymptomatic 11)

A periapical radiograph was taken in the follow-up. Root length formation, increase in dentin thickness, and root end closure was assessed in the radiographs at 06 and 09 months (Fig. 2). At the 12-month follow-up, the patient was asymptomatic, and increased root lengthening, closure of apex, and increased dentinal thickness were noted. The apical closure was confirmed with Cone Beam Computed Tomography (CBCT) (Fig. 4). Pulp canal obliteration was pointed out at the cervical third of the root canal at the end of the 12-month follow-up period. Further, the clinical and radiographic appearance correlated with the desired treatment protocol (Fig. 5).

## Discussion

Regenerative Endodontic Treatment is based on the tissue engineering principle that aims to preserve stem cells with the aid of proper scaffolds and growth molecules. An ideal scaffold is required to create a conducive environment for stem cells to migrate, proliferate and differentiate. Most procedures use apical bleeding from periapical tissue into canal space to form a Blood clot (BC) [13]. Still, quantitative and qualitative approximation of control of blood volume in the chamber is difficult; some patients have clinical challenges to induce periapical bleeding due to the vicinity of the nerve as it poses a risk of nerve injury, especially in mandibular premolars [14, 15].

To overcome the disadvantages of the BC scaffold, platelet concentrates have taken the front line in regenerative procedures and have proven promising, in line with the findings of the current case report. These are autologous and reasonable and consist of substantial growth factors, including (TGF- beta, VEGF) and platelet-derived growth factor (PDGF) [16]. State-of-the-art mechanisms to extract and auto-utilize a hematological derivative have proven to be the source of clinical usage [17]. PRF doesn’t require much chemical preparation as it excludes exogenous bovine thrombin. It is an organized fibrin gel entrapping platelet and growth factors; acts as a biodegradable scaffold that slowly releases growth factors, stimulating wound healing and soft tissue regeneration [18]. In vitro studies have shown great potential in cell attachment and proliferation and help angiogenesis. The difference between a natural blood clot and PRF is that PRF is more homogenous and easier to handle and place in the canal, thus improving clinical applicability [19].

Gaining long-term survival and success in the regeneration of an incompletely developed non-vital tooth in terms of clinical and radiographic parameters was attained in the case, which was attained utilizing constant mechanical plaque control, standardized sterilization protocol, operatory disinfection, and monitoring for persistent infection. Almutairi W et al. (2019) findings were in agreement with the current study as a detailed understanding of dental trauma, the etiology, preoperative variables, postoperative follow-up, and intraoperative standards of the patient are to be maintained to acclaim a novel therapeutic goal [20]. Aligning with the etiology of the present study, Zeng et al. (2022) found that post-operative failure in RET of young permanent teeth with a history of dental trauma might be due to damage in blood supply in the periapical area and reduced resistance to infection [21].

In this case, we have combined the reliability of BC and the promising action of platelet concentrates (PRF), as the combination may yield better results and faster healing than PRF or BC alone [22]. The disinfection of the root canal and stimulation of residual stem cells induce new hard tissue formation on the dentinal wall and results in continued root development [23]. The apical closure began at a six-month follow-up, and complete closure of the apex occurred at 12 months, was confirmed. Dentinal wall thickness increased, and root lengthening was also noted during the follow-up period. The mean success rate of PRF for apical closure after one year was well above 85.1%, and root lengthening at 74.1%. Periapical healing is 100% for PRF and further strengthens the immature tooth; the dentinal wall thickness excess of 1.5 mm, and the tooth will have increased fracture resistance [24].

Two unwanted sequelae during the study were discoloration of the tooth and pulp canal obliteration (Figs. 4 and 5). The bismuth oxide in Mineral trioxide Aggregate is the primary reason for tooth discoloration. The tooth discoloration was specifically noted during the sixth-month follow-up. Similarly, in a study by Song et al. (2017), revascularization-associated Intracanal Calcification (RAIC) was noted in 62.1% of cases, out of which 72.2% were classified as pulp canal obliteration [25]. High cases of RAIC are detected as unfavorable sequelae in patients treated with induced bleeding. Bleeding from periapex may carry periodontal stem cells and bone marrow stem cells; which would get recruited into the canal space. These stem cells are capable of osteogenesis and cementogenesis potential and can result in RAIC [26].

**Fig. 6:**
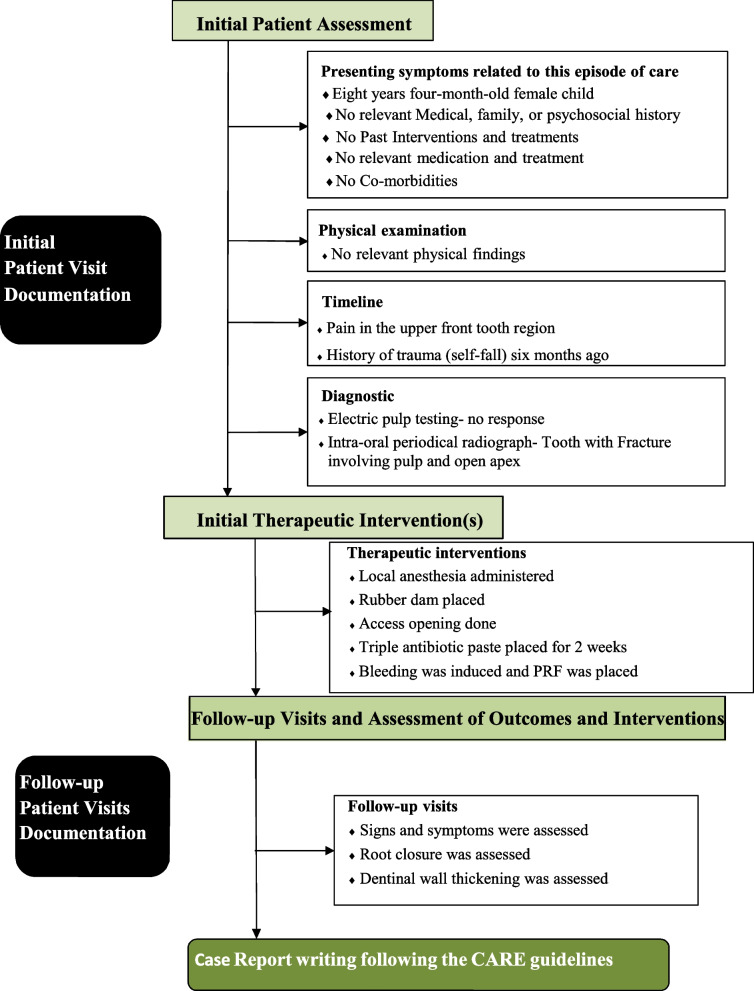
Flow diagram for the Revascularization procedure following the CARE guidelines (adapted from the Equator network)

Regenerative procedures have proved to be successful in restoring damaged pulpal tissue and thus promote assisted healing through neovascularization, growth mediation, and induced matrix development for both soft and hard tissues (Fig. 6).

## Conclusion

Revascularization increases the dentinal wall thickness and allows the closure of the apex in the presented case. RET has proven to be the salient technique to manage long-term nonvital permanent teeth. Large-scale clinical trials would define a standardized protocol based on age, stage of tooth development, anatomical factors, and pathophysiological status. Adequate training, curriculum development, and experience to deliver regenerative therapies are necessary; as suitable skills, technology, and dental tissue regeneration kits should be promoted as a line of treatment to deliver safe, economical, accessible, and established mode endodontic patient management. Considering long-term clinical success, imparting regenerative endodontic training modules in the post-graduate residency program would benefit patients and aid the current era of sustainable dentistry.

## Data Availability

All data underlying the findings and outcome are presented as part of the article and no supplementary source data are required.
